# Magnetic hyperthermia of breast cancer cells and MRI relaxometry with dendrimer-coated iron-oxide nanoparticles

**DOI:** 10.1186/s12645-018-0042-8

**Published:** 2018-10-08

**Authors:** Marzieh Salimi, Saeed Sarkar, Reza Saber, Hamid Delavari, Ali Mohammad Alizadeh, Hendrik Thijmen Mulder

**Affiliations:** 1 Department of Medical Physics and Biomedical Engineering, Faculty of Medicine, Tehran University of Medical Sciences, Tehran, P.O. Box 1417613151, Iran; 2 Research Center for Science and Technology in Medicine, Tehran University of Medical Sciences, Tehran, Iran; 3 Department of Medical Nanotechnology, School of Advanced Technologies in Medicine, Tehran University of Medical Sciences, Tehran, Iran; 4 Department of Materials Science and Engineering, Tarbiat Modares University, Tehran, Iran; 5 Cancer Research Center, Tehran University of Medical Sciences, Tehran, Iran; 6 Department of Radiation Oncology, Erasmus Medical Center Cancer Institute, Rotterdam, The Netherlands

**Keywords:** Magnetic hyperthermia, MRI, Iron-oxide nanoparticles, Dendrimer, Breast cancer

## Abstract

**Background:**

Recently, some studies have focused on dendrimer nanopolymers as a magnetic resonance imaging (MRI) contrast agent or a vehicle for gene and drug delivery. Considering the suitable properties of these materials, they are appropriate candidates for coating iron-oxide nanoparticles which are applied in magnetic hyperthermia. To the best of our knowledge, the novelty of this study is the investigation of fourth-generation dendrimer-coated iron-oxide nanoparticles (G_4_@IONPs) in magnetic hyperthermia and MRI.

**Methods:**

IONPs were synthesized via co-precipitation and coated with the fourth generation (G_4_) of polyamidoamine dendrimer. The cytotoxicity of G_4_@IONPs with different concentrations was assessed in a human breast cancer cell line (MCF_7_) and human fibroblast cell line (HDF_1_). Hemolysis and stability of G_4_@IONPs were investigated, and in addition, the interaction of these particles with MCF_7_ cells was assessed by Prussian blue staining. Heat generation and specific absorption rate (SAR) were calculated from measurement and simulation results at 200 and 300 kHz. MCF_7_ and HDF_1_ cells were incubated with G_4_@IONPs for 2 h and then put into the magnetic coil for 120 min. Relaxometry experiments were performed with different concentrations of G_4_@IONPs with T1- and T2-weighted MR images.

**Results:**

The TEM results showed that G_4_@IONPs were 10 ± 4 nm. The in vitro toxicity assessments showed that synthesized nanoparticles had low toxicity. The viability of MCF_7_ cells incubated with G_4_@IONPs decreased significantly after magnetic hyperthermia. In addition, MR imaging revealed that G_4_@IONPs improved transverse relaxivity (r2) significantly.

**Conclusions:**

Our results encouraged the future application of G4@IONPs in magnetic hyperthermia and MR imaging.

## Background

Recently, magnetic hyperthermia is increasingly being used in cancer treatment due to its advantages over other treatments (Jiang et al. 2014). In magnetic hyperthermia, an alternating magnetic field (AMF) is utilized to heat magnetic nanoparticles (MNPs) such as iron-oxide nanoparticles (IONPs), which increases the tumor temperature by 3–7 ^°^C (Pearce et al. 2013; Prasad et al. 2007). Samanta et al. assessed the thermoablation efficiency of protein-coated iron-oxide NPs (size = 12.1 nm ± 1.6 nm) in cultured HeLa cells incubated with MNPs (4 mg/ml) for 2 h followed by 45 min of AMF exposure (400 kHz, 6.3 kA m^−1^, SAR = 36 W/g_Fe_). Immediately after treatment, cytotoxicity was measured to show demolition of cancer cells due to MNPs heating (Samanta et al. 2008).

MRI has been applied as a common medical diagnostic instrument due to its noninvasive nature and topographic properties. This technique is based on the magnetic relaxation of water protons under an external magnetic field (Kojima et al. 2011). IONPs can also be used as MRI contrast agents because of their unique properties including high chemical activity, biocompatibility, and saturation magnetization (Kojima et al. 2011; Barick et al. 2012; Khot et al. 2013).

In this study, IONPs were coated with dendrimers, which are a promising category of coating materials and have attracted significant attention in recent years (Tajabadi et al. 2013). Indeed, dendrimers, compared to the other nanovectors including micelles and liposomes, whose properties are difficult to control, have tunable characteristics which make them attractive materials for biomedical applications (Tomalia et al. 2007). PAMAM dendrimers are hydrophilic, biocompatible, monodisperse, and high-branched three-dimensional macromolecules with void spaces in their internal structure (Mohammad and Yusof 2014; Wolinsky and Grinstaff 2008). On the other hand, dendrimer-based contrast agents possess sufficient binding sites to which numerous imaging, targeting, and therapeutic moieties can be conjugated (Longmire et al. 2008). In the 1990s, Lauterbur et al. demonstrated the feasibility of dendrimer-based MRI contrast agents for the first time in vascular imaging (Tomalia 2006). Chandra et al. indicated that dendrimers loaded with doxorubicin improved the efficiency of this drug for demolishing breast cancer cells and recommended this nanostructure for combined cancer therapies (Chandra et al. 2011). Given the importance of using coating materials with the capability of being used in theragnostic applications, dendrimers were selected as IONPs coating in this study.

To the best of our knowledge, no research has been performed on the application of G_4_@IONPs in magnetic hyperthermia. This study was performed to investigate the efficiency of G_4_@IONPs in magnetic hyperthermia of breast cancer cells and the potential of using G4@IONPs as an MRI contrast agent by determining the relaxivity of this nanostructure with MRI.

## Methods

### Materials

Ferric sulfate heptahydrate (FeSO_4_·7H_2_O, 99% w/w), ferric chloride hexahydrate (FeCl_3_, 6H_2_O, 99% w/w), hydrochloric acid (HCl, 32% v/v), ammonia solution (NH_3_, 32% v/v), methanol (99.9% v/v), 3-aminopropyltriethoxysilane [NH_2_(CH_2_)_3_-Si-(OCH_3_)_3_, APTS], ethanol (99.9% v/v), methyl acrylate (99.5% v/v), methoxy-PEG and ethylenediamine (99% v/v), Eagle’s minimal essential medium (DEMEM), fetal bovine serum (FBS), and PenStrep were used in synthesis process and cell culture; all materials were purchased from Sigma-Aldrich (Germany).

### Synthesis of IONPs and APTS-coated IONPs

IONPs were synthesized via the co-precipitation method which is explained in our previous study (Salimi et al. 2018). Briefly, 0.84 g of FeSO_4_ and 1.22 g of FeCl_3_ were dissolved in 20 ml deionized water followed by 30 min sonication. Then, 1 ml of 2 M HCl was slowly added with vigorous stirring in a nitrogen atmosphere. After 2 min, 4.6 ml ammonia was quickly added to the solution and stirring was continued for 1 min. The black precipitate of IONPs was washed five times with distilled water and ethanol through magnetic decantation. To IONPs coating with APTS, 150 ml ethanol was added to 25 ml of 5 g l^−1^ IONPs solution which was sonicated for 30 min; after 20 min of sonication, 300 µl of APTS was added to the mixture. Finally, the solution was stirred for 15 h at room temperature, and eventually, the resultant black precipitate was washed with ethanol three times (Khot et al. 2013).

### Surface coating of IONPs with dendrimer and PEGylation

10 ml ethanol was added to the APTS@IONPs solution after 30 min sonication; subsequently, methyl acrylate/methanol solution (20%, v/v) was added (50 ml) at 0 °C during the sonication for 1 h followed by stirring for 48 h. Then, after washing the resultant solution with methanol, 15 ml ethylenediamine/methanol (50%, v/v) was added and the solution was sonicated for 3 h at 25 °C. This process was repeated to earn the fourth dendrimer generation (G_4_). The final solution was washed several times with methanol and water by magnetic decantation or centrifugation (Khodadust et al. 2013).

Eventually, mPEG molecules (molecular weight of 4000 Da) were conjugated to the surface of amino groups of dendrimers. The applied mPEG mass was three times more than the mass of iron. The mPEG was dissolved in the ethanol solution and added to the G_4_@IONPs solution followed by 18 h reflux.

### Characterization

The morphology and size distribution of G_4_@IONPs were studied by transmission electron microscopy (TEM) and the hydrodynamic size and surface potential were measured through dynamic light scattering (DLS) and zeta potential, respectively. Magnetic properties of IONPs and G_4_@IONPs were measured by vibrating sample magnetometer (VSM) at 300 K under the magnetic field up to 15 kOe. Furthermore, the crystalline phase of IONPs was confirmed by X-ray diffraction (XRD, *λ* = 0.15406 nm) and G4 PAMAM bonds on the surface of IONPs was detected by Fourier-transform infrared spectroscopy (FTIR).

### Cytotoxicity assay

MTT (3-[4,5-dimethylthiazol-2yl]-2,5-diphenyltetrazolium bromide) assay was used to determine the cytotoxicity of G_4_@IONPs in MCF_7_ and HDF_1_ cell lines. Cells were incubated in 96-well plates at a cell density of 4 × 10^3^ cells per well and cultured in DMEM supplemented with 10% FBS and 1% PenStrep at 37 °C and 5% CO_2_ for 24 h. Subsequently, cells were washed twice with PBS and treated with serum-free culture media containing G_4_@IONPs in concentrations of 1500, 1000, 500, 100, 10, 1, and 0 (control) µg/ml. After incubation for 24 h, the culture media were removed, and 100 µl of serum-free medium and 10 µl of MTT solution were added to each well for 4 h. Finally, 100 µl of dimethyl sulfoxide (DMSO) was added; the absorbance of wells was measured using ELISA reader (Hiperion, microplate reader MPR4+) at 540 nm (Salimi et al. 2013).

### Hemolysis assay

Blood samples from healthy male BALB/c mice were collected in heparin-coated tubes. The red blood cells (RBCs) were obtained by centrifuging the blood samples at 1500 rpm for 5 min and removing the upper plasma. The RBCs were washed three times with sterile isotonic 0.9% NaCl solution, and the purified RBCs were suspended in sterile isotonic 0.9% NaCl. The RBC suspension (300 µl) was mixed with 1 ml of 1000, 500, 250, 100, 50, and 10 µg/ml of G_4_@IONPs. All samples were incubated at 37 ^°^C for 2 h and then centrifuged for 2 min at 4000 rpm. Distilled water and isotonic 0.9% NaCl were applied as positive and negative controls, respectively. The supernatant absorbance was measured at 540 nm using ELISA reader (Hiperion, microplate reader MPR4+) (Li et al. 2015). The hemolysis percentage was calculated using the following equation:

1 $${\text{\% Hemolysis}} = \frac{{{\text{OD}}_{\text{sample}} - {\text{OD}}_{\text{negativecontrol}} }}{{{\text{OD}}_{\text{positivecontrol}} - {\text{OD}}_{\text{negativecontrol}} }} \times 100.$$

### Stability of G_4_@IONPs

The stability of G_4_@IONPs was investigated by recording the change in turbidity in 50% FBS. 150 µl of G_4_@IONPs suspension (100 µg/ml) was added to 150 µl of FBS in 96-well plates and incubated for different times up to 72 h at 37 °C. After that, the absorbance of samples was measured at 405 nm. A solution of 5% glucose was employed as a negative control (Li et al. 2015).

### Temperature–time curves

The radiofrequency (RF) absorption of G_4_@IONPs was determined by establishing the AMF-specific absorption rate (SAR), which is defined as the amount of induced heat per unit mass of MNPs per unit of time ($\frac{\Delta T}{\Delta t}$) (Wolinsky and Grinstaff 2008; Xia et al. 2009). A magnetic hyperthermia research system (LABA, HT-1000W, NATSYCO) with a frequency range of 100–400 kHz was used. An Eppendorf microtube containing G_4_@IONPs solution (200 µl) was inserted inside the water-cooled magnetic induction copper coil (6 cm in diameter). The temperature rise was measured with a digital thermometer and plotted against time (temperature–time curve) at frequencies of 200 and 300 kHz with a field intensity of 12 kA/m. The SAR values were calculated using the following equation:

2 $${\text{SAR}} = \left( {\frac{1}{{m_{\text{Fe}} }}} \right)C\left[ {\frac{\Delta T}{\Delta t}} \right],$$

where *m*_Fe_ is the mass of iron in the sample, *C* is the specific heat capacity of the sample, and $\left[ {\frac{\Delta T}{\Delta t}} \right]$ is the initial slope of the temperature–time curve (Natividad et al. 2008, 2009). The net temperature change was yield by the following equation:

3 $$\Delta {\text{T}} = {\text{T}}_{\text{n}} - {\text{T}}_{0} ,$$

where *T*_0_ and *T*_n_ are initial temperature and temperature at the interval, respectively. The SAR was estimated from the initial and steepest part of the slope of the time–temperature curve. We determined the appropriate interval for calculating the slope by analyzing the plot of incremental temperature change and selecting the region with a constant first derivative of the heating rate. The temperature change was calculated over every interval (i.e., *T*_n_ − *T*_n−1_), and the results were plotted versus heating time (*t*) (Bordelon et al. 2011).

### Simulation of heat generation and transfer

To verify the measurement results, simulation of heat generation due to magnetic hyperthermia was performed using COMSOL Multiphysics. The microtube, MNPs solution, and surrounding atmosphere were implemented to assess the heat transfer in a time-transient manner. Different heat transfer mechanisms were included in the simulations, e.g., heat transfer in fluids for the solution and surrounding air, and heat transfer in solids for the tube. Based on certain values of SAR for 1 ml of G_4_@IONPs suspension, heat generation and heat transfer rates were modeled as functions of time.

### Histochemistry analysis

For Prussian blue staining, used to detect the presence of iron, MCF_7_ cells were incubated in a medium containing G_4_@IONPs (500 µg/ml) for 2 h. Subsequently, cells were fixed with 4% formalin at room temperature for 20 min and washed with PBS, followed by the incubation with 10% potassium ferrocyanide in 10% HCl (50%, v/v) for 20 min (Samanta et al. 2008). MCF_7_ cells after 2 h incubation with 500, 250, 100, 50, and 0 (control) μg/ml G_4_@IONPs were trypsinized and collected by centrifugation. The collected cells were lysed by 2 ml 65% nitric acid. The amount of the nanoparticles cell uptake was quantified using inductively coupled plasma mass spectrometry (ICP-MS) (Varian Inc, Palo Alto, CA) and the resulting concentration was divided by counting the cell numbers.

### Magnetic hyperthermia in breast cancer and normal cells

At 24 h after seeding 4 × 10^5^ cells (MCF_7_ and HDF_1_) in a 35 mm dish, cells were incubated with medium with/without the G_4_@IONPs in a concentration of 500 μg/ml for 2 h at 37 °C. For hyperthermia treatment, these cells were put in the magnetic coil for 120 min (12 kA/m and 300 kHz), and control cells were left in the incubator at 37 °C. Immediately afterward, the viability of cells was assessed by MTT assay.

### Apoptosis assay

After hyperthermia condition, identifying of apoptotic cells was determined using in situ cell death detection kit (Roche, Mannheim, Germany) terminal uridine deoxynucleotidyl transferase dUTP nick end labeling (TUNEL) staining. Assay performed according to the manufacturer’s protocol. Briefly, MCF_7_ cells were fixed by 4% paraformaldehyde for 10 min, permeabilized with 0.2% Triton X-100 for 2 min on ice, and incubated with a mixture of TUNEL reaction. Cells treated with 5% ethanol for positive apoptosis control. For negative apoptosis control, cells were induced only with label solution. Evaluation performed by an inverted fluorescent microscope.

### Relaxivity measurements of G_4_@IONPs

Relaxivity is a measure of the ability of a contrast agent to enhance the relaxation of adjacent hydrogen spins, which can improve contrast in MRI images (Barick et al. 2014). To assess the relaxivity of G_4_@IONPs, samples were prepared at different concentrations of 1, 2, 4, 8, 10, 12, 16, and 20 µg/ml and placed in a plastic container. Longitudinal and transverse relaxation times (*T*_1_ and *T*_2_) were measured by 3 T MRI scanner (Trio Tim/SIEMENS, Munich, Germany). To measure *T*_1_, 6 spin echoes (SE) images were acquired with an echo time (TE) of 12 ms and repetition times (TR) of 3000, 2000, 1000, 500, 250, and 100 ms. To measure *T*_2_, 32 SE images were obtained with TR of 3000 ms and TE of 12–384 ms. The signal intensity (SI) of each concentration was determined via RadiAnt Dicom viewer software and calculated by Eqs. 4 and 5:

4 $${\text{SI}} = S_{0} \left( {1 - {\text{e}}^{{\frac{{ - {\text{TR}}}}{{T_{1} }}}} } \right)$$

5 $${\text{SI}} = S_{0} {\text{e}}^{{\frac{{ - {\text{TE}}}}{{{\text{T}}_{2} }}}} ,$$

where R_1_ and R_2_ curves were obtained via logarithmic fitting to SI versus TR and TE curves, respectively, using the OriginPro 2016 software. Eventually, the relaxivity values (*r*_1_ and *r*_2_) were estimated using a linear fit to R_1_ and R_2_ versus G_4_@IONPs concentration curves, respectively. All sequences were acquired using a 280 × 280 mm^2^ field of view (FOV), a resolution of 256 × 230 pixels, and slice thickness of 7 mm.

To evaluate the in vivo capability of G_4_@IONPs, an MRI study was performed on animal models. BALB/c mice were intravenous injected with 0.2 ml G_4_@IONPs at Fe concentration of 1 mg/ml. The MRI experiments were performed using a 3 T MRI scanner (MAGNETOM Prisma/SIEMENS, Munich, Germany) with a magnetic field intensity of 3 T. The following parameters were adopted for obtaining in vivo MR images and signal intensity analysis in vivo: FOV 6 × 6 cm, matrix size = 256 × 125, slice thickness 4 mm, TEs 41.6, 71, 113.6 ms, and TR 2000 ms.

### Statistical analysis

All data were expressed as mean ± SD and one-way ANOVA was used for statistical analysis. *P* < 0.05 was considered statistically significant.

## Results

### Characterization of G_4_@IONPs

**Fig. 1:**
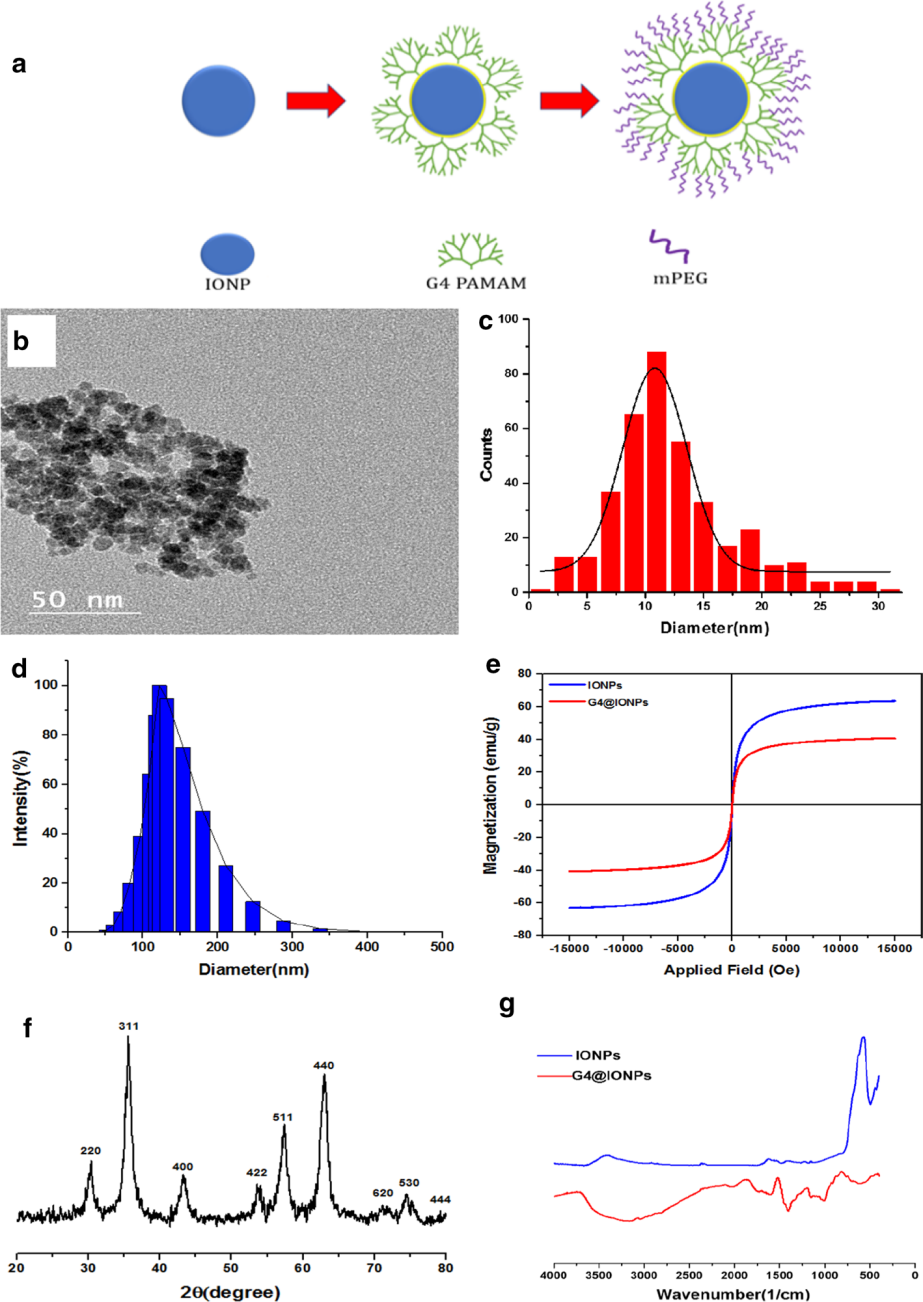
**a** Schematic of G_4_@IONPs synthesis, **b** TEM image, and **c** size distribution of G_4_@IONPs, **d** DLS shows hydrodynamic size of G_4_@IONPs, **e** VSM illustrates the maximum magnetizations of IONPs and G_4_@IONPs at room temperature, **f** XRD identifies the crystal structure and shows all diffraction peaks assigned to IONPs, and **g** FT-IR spectra of pure IONPs and G_4_@IONPs

The schematic of G4@IONPs synthesis is shown in Fig. 1a. The size of G_4_@IONPs was 10 ± 4 nm measured by TEM (Fig. 1b, c). Hydrodynamic size of G_4_@IONPs was 120 nm as measured by DLS, and surface potential of these NPs was +35 mV at PH = 7 and 25 °C (Fig. 1d). M–H curve without any hysteresis loop was measured above the blocking temperature by VSM and maximum magnetization for IONPs and G4@IONPs at room temperature were 63.4 and 40.6 emu g^−1^, respectively (Fig. 1e). Results of XRD indicated that all diffraction peaks could be assigned to Fe_3_O_4_ without any impurities (Fig. 1f). The XRD pattern did not change by coating the NPs with PAMAM dendrimers (Khodadust et al. 2013). In FTIR spectra, the presence of the Fe_3_O_4_ core could be detected by the strong peaks between 408 and 673 cm^−1^; as well as the magnetite Fe–O group bond observed at 570 cm^−1^, corresponding to the intrinsic stretching vibration of the metal at the tetrahedral site (Fe tetra–O) (Julian JM, Brezinski DR. An infrared spectroscopy atlas for the coatings industry 1991). The broad peak at 3444 cm^−1^ exhibited the bending mode of free NH_2_ groups present at APTS (Yamaura et al. 2004). The peaks of –CO–NH– bonds were detected at 1490, 1570, and 1620 cm^−1^ (Fig. 1g) (Tsubokawa and Takayama 2000).

### Colloidal stability (aggregation) of G_4_@IONPs

**Fig. 2:**
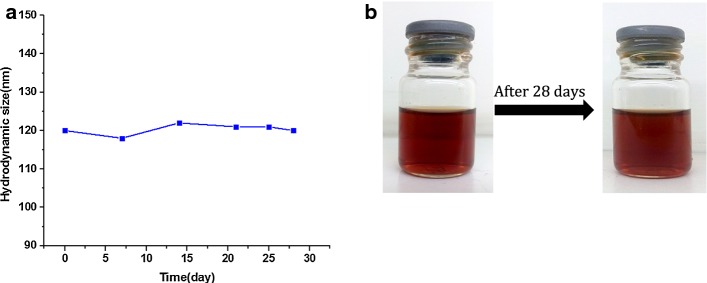
**a** Hydrodynamic sizes of G_4_@IONPs in aqueous solution as a function of storage time. **b** Time evolution of the sedimentation photographs of freshly prepared G_4_@IONPs solution (left) and the solution after 28 days

It is imperative to consider that the colloidal stability of G_4_@IONPs should be investigated before MRI applications. The hydrodynamic size maintained in a narrow range by DLS measurement after at least 28 days (almost 1 month), as shown in Fig. 2a. There was no sign of aggregation and flocculation or subsidence after 28 days of storage (Fig. 2b).

### Cytotoxicity assay

**Fig. 3:**
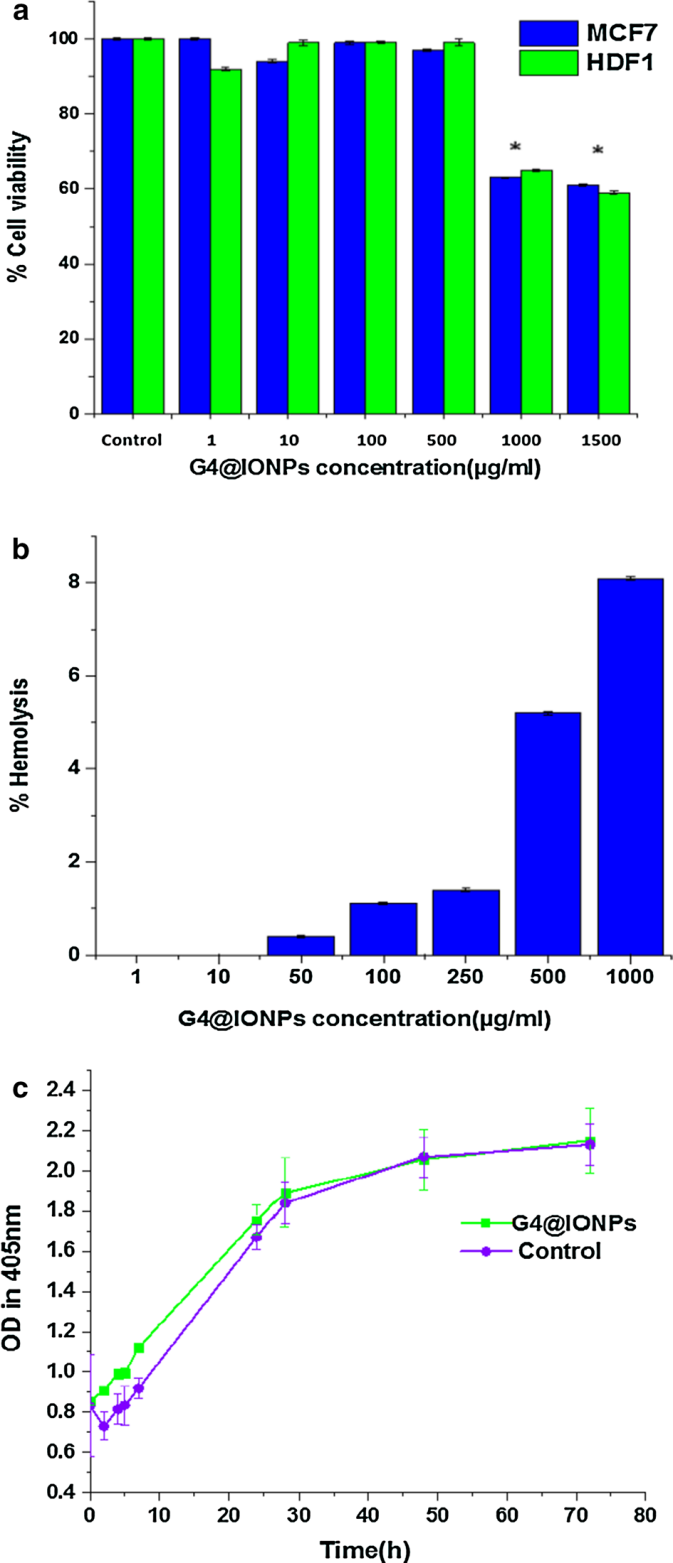
**a** Cytotoxicity of G_4_@IONPs in MCF_7_ and HDF_1_ cells (*P < 0.05). The cytotoxic effect at concentrations of 1000 and 1500 µg/ml is significant in both cell lines. **b** Hemolysis assay was fulfilled to assess the effect of G_4_@IONPs on RBCs. **c** No significant increase in turbidity of G_4_@IONPs suspension was detected after different incubation times

The MTT assay was performed to investigate cytotoxic effect of G_4_@IONPs on MCF_7_ and HDF1 cell lines at concentrations of 1500, 1000, 500, 100, 10, 1 µg/ml and 0 (control). The results clearly indicated that G_4_@IONPs had no significant cytotoxicity at concentrations of 500 µg/ml and lower. At 1000 and 1500 µg/ml, however, the viability of both cell lines decreased significantly (for MCF_7_:63% and 61%; for HDF_1_: 65% and 59%, respectively) (Fig. 3a).

### Hemolysis and stability assessments

Hemolysis assay was performed to assess the effect of G_4_@IONPs on RBCs (Feng et al. 2014). The outcomes showed that G_4_@IONPs at all concentrations did not have any hemolysis effect; the highest hemolysis activity was only 8.1% at 1000 µg/ml (Fig. 3b).

To investigate the stability of G_4_@IONPs in a biological environment, the turbidity assay was performed via measuring the turbidity changes of G_4_@IONPs suspension in FBS. No significant difference in turbidity of G_4_@IONPs and control suspensions was detected over the different incubation times; as the turbidity changes in both suspensions were similar after 72 h of incubation (Fig. 3c).

### Temperature–time curves and simulation

**Fig. 4:**
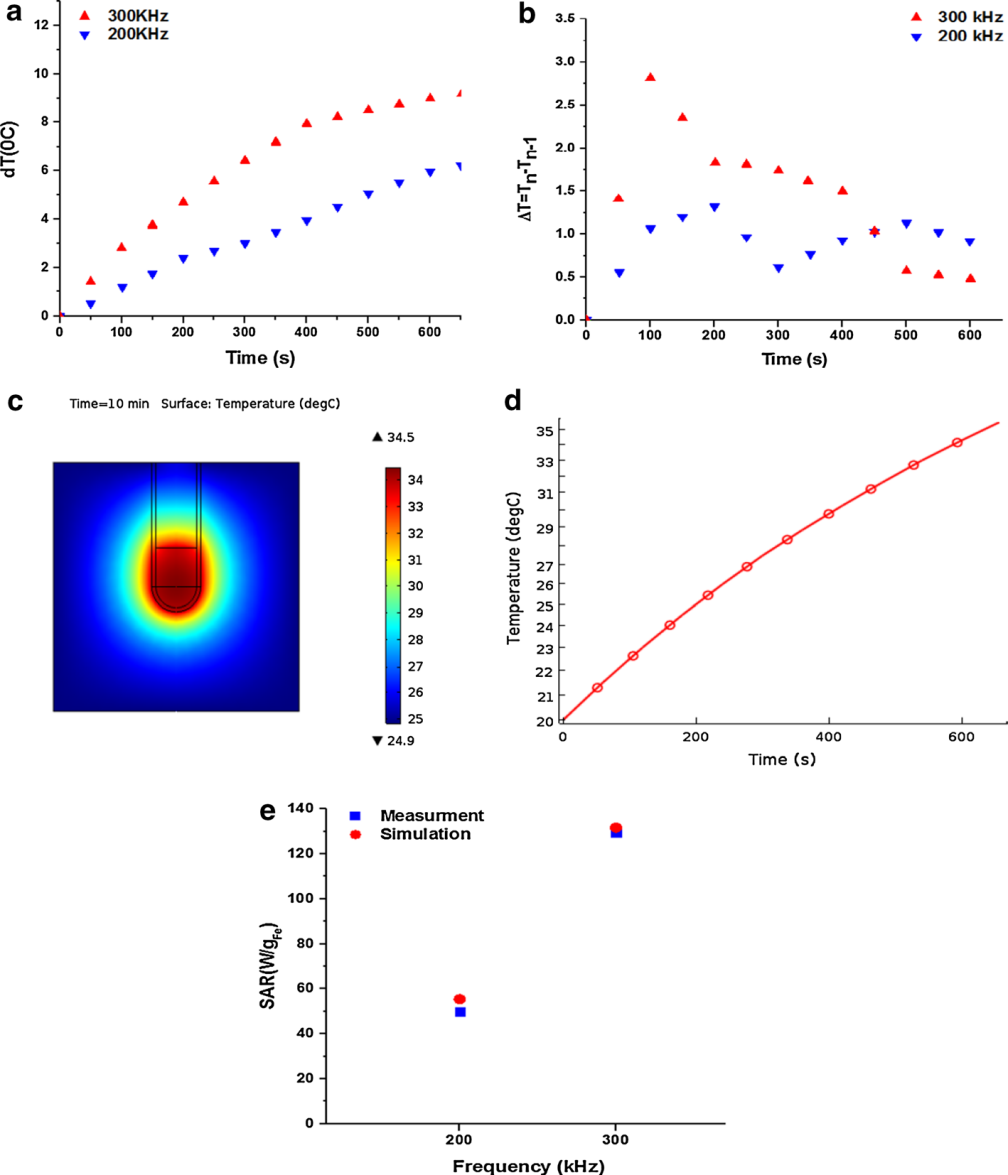
**a** Temperature–time curves at 200 and 300 kHz. **b** Incremental temperature changes, *T*_n_ − *T*_n−1_ over time. **c** Temperature distribution in the cross section of the model structure. **d** Simulated temperature–time curve at 300 kHz after 10 min. **e** SAR values of the measurements and numerical analysis results at frequencies of 200 and 300 kHz

Figure 4a illustrates temperature–time curves at 200 and 300 kHz during AMF exposure. The incremental heating curve was drawn, and the time interval with a constant non-zero value was used to find the area in the temperature–time curve with a constant slope (Fig. 4b); since in area, there was not any temperature decay in the sample. The temperature distribution in the cross section of the model structure and temperature–time curve at 300 kHz after 10 min is shown in Fig. 4c, d. The SAR calculated from measurements at 200 and 300 kHz were 49.8 and 129.3 W/g_Fe_, respectively; the results were compared to the calculated SAR values in a simulation. The SAR values of the measurements were in a good agreement with the numerical analysis results (Fig. 4e).

### Internalization of G_4_@IONPs into cells

**Fig. 5:**
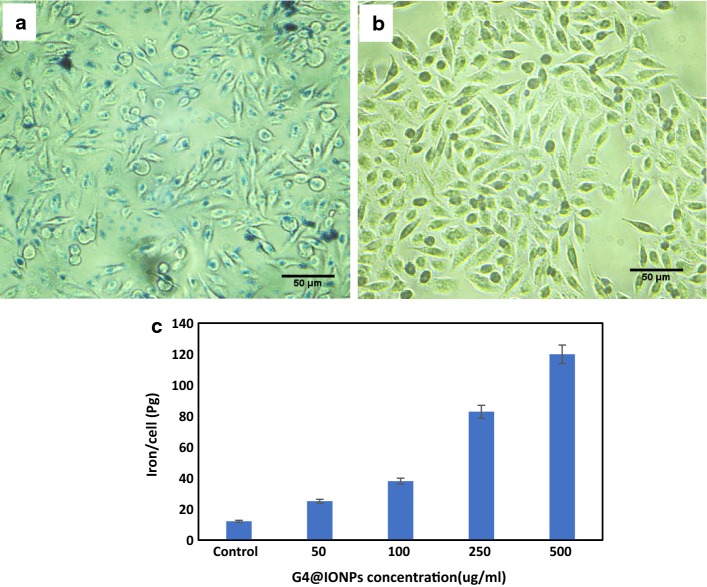
MCF_7_ cells incubated **a** with and **b** without (control) G_4_@IONPs followed by Prussian blue staining. **c** ICP-MS of different concentrations of G_4_@IONPs

Cell internalization of G_4_@IONPs assessed by Prussian blue staining illustrated a high density of iron inside the MCF_7_ cells after 2 h incubation with G_4_@IONPs. The iron appeared as the blue precipitate in the cytoplasm (Fig. 5a, b). The results of ICP-MS showed that higher amount of iron (119.8 ± 3.5 pg) was taken up in higher G_4_@IONPs concentration (500 μg/ml) (concentration-dependent). After identical sample preparation in the control, 12.1 ± 2.7 pg iron per cell could be detected. In the case of 50, 100, and 200 μg/ml of G4@IONPs, the numbers were 25, 38, and 82.8 pg/cell, respectively (Fig. 5c).

### Cell viability affected by magnetic hyperthermia

**Fig. 6:**
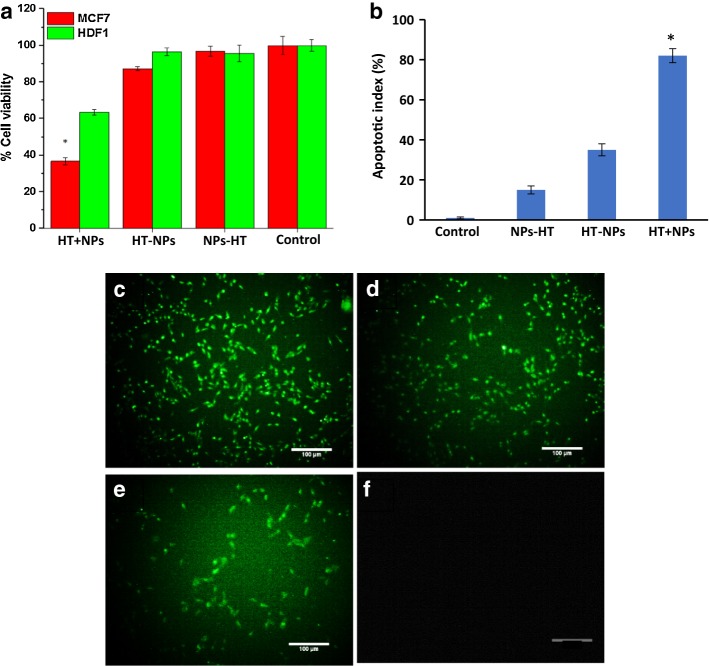
**a** MCF7 and HDF1 cells’ viability percentage after magnetic hyperthermia (*P < 0.05); **b** apoptotic index (*P < 0.05); TUNEL staining illustrated the apoptotic MCF_7_ cells in groups of: **c** HT+NPs **d** HT-NPs **e** NPs-HT, and **f** control

Immediately after magnetic hyperthermia (HT), the viability of MCF_7_ and HDF_1_ cells was assessed in all groups (HT + NPs, HT − NPs, NPs − HT, and control) by MTT assay. Cell viability percentage in the HT + NPs group decreased significantly (36.7 ± 2%), while the viability in other groups did not significantly decrease compared with control group (96.8 ± 2.3% and 87.3 ± 1.12%, respectively). The viability of HDF_1_ cells cultured with G_4_@IONPs did not decrease significantly due to AMF exposure (63.5 ± 1.5%) (Fig. 6a).

### TUNEL assay

Results of TUNEL assay showed the apoptosis of breast cancer cells immediately after magnetic hyperthermia in all groups (HT + NPs, HT − NPs, NPs − HT, and control) (Fig. 6b). The results indicated apoptotic cells in HT + NPs were higher than that in other groups (82%), while the fewer apoptosis was seen in groups of HT − NPs and NPs − HT (35% and 15%, respectively) (Fig. 6c–f).

### MRI relaxometry and in vivo images

**Fig. 7:**
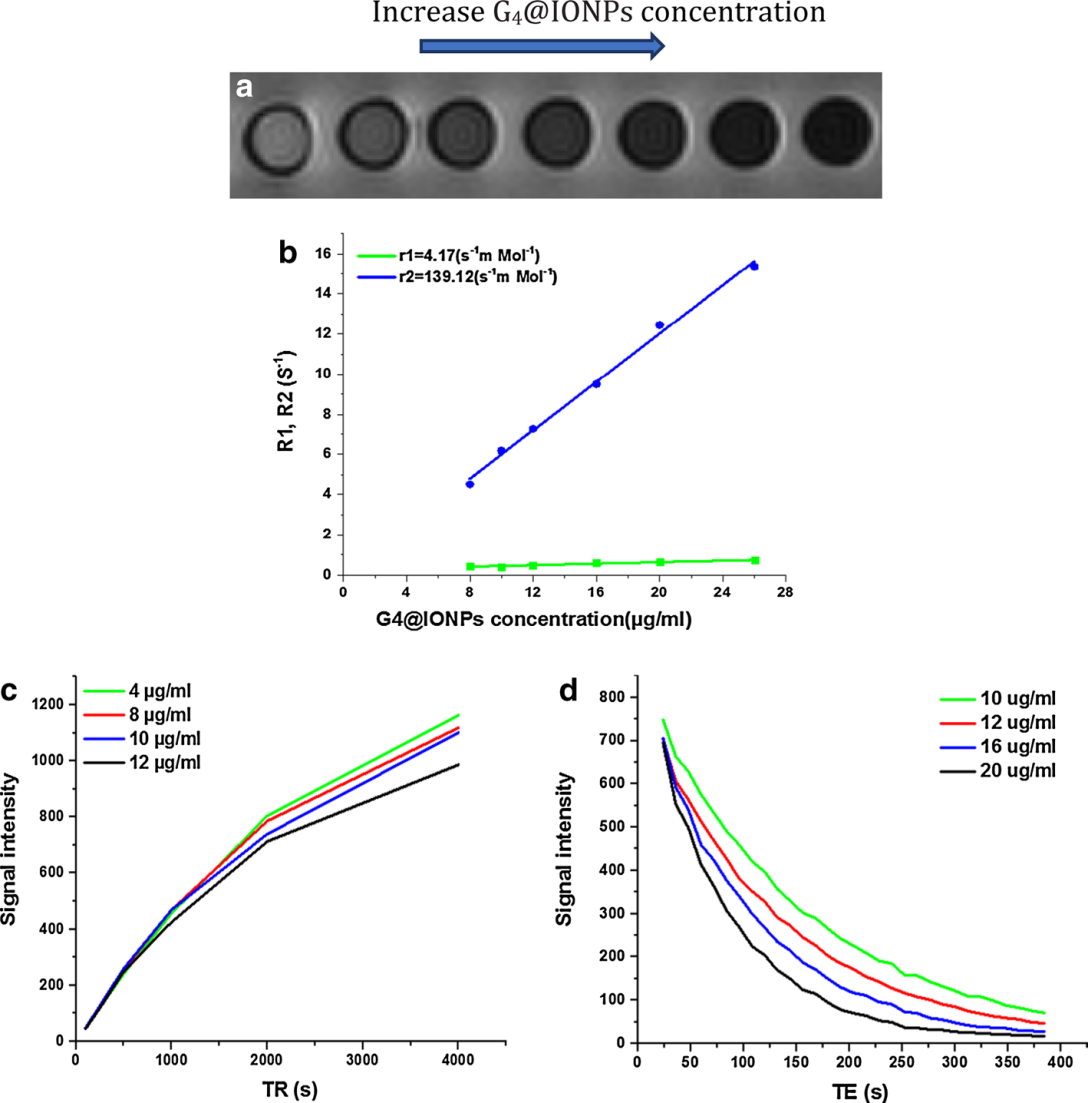
**a** T_2_-weighted MR images of G_4_@IONPs as a function of IONPs concentration, **b** relaxation rates (*R*_1_, *R*_2_) versus concentration of G_4_@IONPs, **c** increasing the SI versus TRs at different concentrations of G_4_@IONPs, and **d** decreasing the SI versus TEs at different concentrations of G_4_@IONPs

Multi-echo SE images of G_4_@IONPs showed decreasing signal intensity with increasing iron concentration (Fig. 7a). Indeed, the signal loss was more in higher iron concentrations because of shortening T_2_. R_1_ and R_2_ relaxation curves versus G_4_@IONPs concentration are depicted in Fig. 4b. Longitudinal (*r*_1_) and transverse (*r*_2_) relaxivities extracted from *R*_1_ and *R*_2_ curves were 4.17 s^−1^Mm^−1^ and 139.12 s^−1^Mm^−1^, respectively (Fig. 5b). Due to the T_1_ effect, signal curves versus TRs increased by decreasing G_4_@IONPs’ concentration (Fig. 7c), while signal curves versus different TEs decreased with increasing the G_4_@IONPs’ concentration due to T_2_ relaxation time (Fig. 7d).

**Fig. 8:**
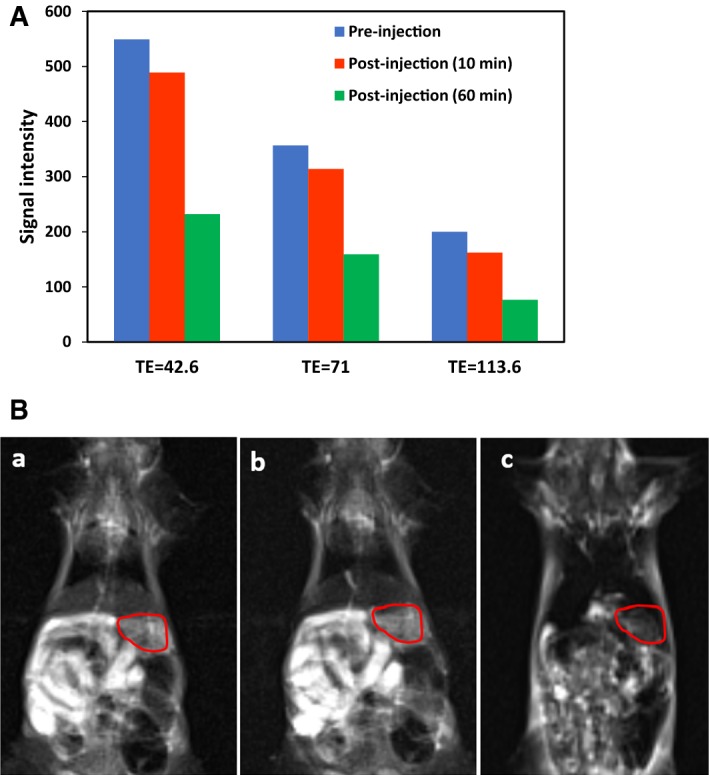
**A** In vivo MR signal intensity of liver (TR = 2000 and different TEs) before, 10 and 60 min after intravenous injection of G_4_@IONPs; **B** In vivo MR images of BALB/c mice (**a**) before, and after (**b**) 10 min and (**c**) 60 min intravenous injection of G_4_@IONPs (TR = 2000, TE = 71). The red contours show the liver tissue

The capability of G_4_@IONPs to visualize liver tissues was evaluated in male BALB/c mice. As shown in Fig. 8A, we acquired MR images with different TEs before, 10 and 60 min after intravenous injection. The G_4_@IONPs darkened the MR images of liver with an intravenous injection after 10 and 60 min compared with that before injection; in addition, SI decreased with increasing the TE. It could also be seen that 60 min after injection of G_4_@IONPs, the liver images became substantially darker (more signal intensity) in comparison with acquisition at 10 min post-injection (SI = 232 versus 489 at TE = 42.6), as shown in Fig. 8B (a, b, and c).

## Discussion

The aim of this study was to investigate the application of G_4_@IONPs in magnetic hyperthermia of MCF_7_ cancer cells and also to assess their feasibility as an MRI contrast agent. The G_4_@IONPs were synthesized by co-precipitation and coated with G_4_ PAMAM dendrimer by a stepwise method. The characteristics of G_4_@IONPs are important in determining their behavior in biomedical applications. TEM image showed that the size of G_4_@IONPs was 10 ± 4 nm with a positive surface charge of + 35 mV (cationic) which can be effective on NPs uptake into cultured cells (Mecke et al. 2005).

The cell internalization was assessed by Prussian blue staining. The qualitative results showed that only 2 h after incubation, G_4_@IONPs could enter the MCF_7_ cytoplasm, demonstrated by the blue spots in Fig. 5a. Hong et al. studied the interactions of PAMAM dendrimers and KB and Rat2 cell membranes (Hong et al. 2006). Their results showed that these polymers induced the formation of transient, nanoscale holes in living cells which allowed a significantly increased exchange of materials across the cell membrane. The authors claimed that the size of the polymers did not seem to markedly affect their ability to induce hole formation in the membranes. Furthermore, the architecture (sphere-like) of PAMAM polymers was effective for hole formation and increasing membrane permeability. Seib et al. also assessed the effect of generation (G_2_–G_4_) of PAMAM dendrimer on the endocytic capture and intracellular entrance by flow cytometry (Seib et al. 2007). They showed that all the cationic polymers were internalized via endocytosis, with maximum uptake for cationic G_4_ PAMAM.

In general, MNPs heating in magnetic hyperthermia are due to three independent mechanisms including hysteresis loss, Brownian and Néel relaxation (Deatsch and Evans 2014; Mamiya and Jeyadevan 2011). The Brownian mode represents the rotational friction component in a given suspending medium. As the whole particle oscillates towards the magnetic field, the suspending medium opposes this rotational motion resulting in heat generation. The Néelian relaxation represents the rotation of the individual magnetic moments towards the alternating field (Rosensweig 2002).

When MNPs are exposed to an AMF, the magnetic moments tend to align in the direction of the field; in MNPs exhibiting hysteresis, domain walls can then move in the presence of an applied magnetic field; as a result, many single domains combine and create larger domains (domain growth). This shifting of domain walls produces the heat and continues until the point of magnetic saturation (Etheridge et al. 2012). Considering the VSM result, the G_4_@IONPs did not have any hysteresis loop, meaning that the heating was only due to Brownian motion and Néelian relaxation (Deatsch and Evans 2014).

The hydrodynamic diameter was larger than that measured by TEM that might be because of two main reasons; first, the scattering of small particles varies strongly with particle radius in DLS and also the signal of larger particles can still overwhelm the signal of smaller ones. The second reason is that the techniques based on light scattering like DLS require a higher concentration of NPs solution which is more likely to lead to aggregation. Consequently, these aggregated NPs have a disproportionate influence on the analytical signal (Kim et al. 2014; Domingos et al. 2009). The colloidal stability results showed that there was not any aggregation or change in hydrodynamic diameter of G_4_@IONPs after 28 days; it revealed that these MNPs had great colloidal stability which is a prerequisite for biomedical applications.

Furthermore, the hydrodynamic diameter (*V*_H_) is crucial in Brownian relaxation time (τ_*B*_): τ_*B*_=3*ηV*_H_/*K*_*B*_T, where *η* is the viscosity of medium, *K*_B_ is the Boltzmann constant, and *T* is the temperature (Rosensweig 2002). Indeed, the amount of heat generated due to Brownian relaxation depends inversely on hydrodynamic size (Deatsch and Evans 2014).

The application of IONPs in magnetic hyperthermia depends on the heating efficiency of these particles which is quantified as SAR. In our study, temperature rising measurements were performed at frequencies of 200 and 300 kHz and a field intensity of 12 kA/m. There are several parameters which affect induced SAR, including size, coating, aggregation of MNPs, frequency, and intensity of AMF (Li et al. 2015). SAR calculated from heat measurements was 49.8 and 129.3 W/g_Fe_ at 200 and 300 kHz, respectively; basically, SAR increases with increasing frequency of AMF (Kalber et al. 2005). The simulated SAR values were in a good agreement with measurements. The temperature rising curve at 300 kHz had a plateau at approximately 400 s, which meant a thermal equilibrium between the MNPs sample and the environment was reached at this time. As the plateau after 400 s in the simulation was not as pronounced as in the measurements, this could be due to thermal insulation and leakage of generated heat from sample to environment.

The MTT assay revealed that the cytotoxicity of G_4_@IONPs was negligible at concentrations of 500 µg/ml and below. This could be due to the dendrimer coating and PEGylation, which made IONPs more biocompatible (Kojima et al. 2011). As mentioned before, dendrimers with cationic surface groups tend to interact with the lipid bilayer, increase the permeability, and decrease the integrity of the biological membrane. This causes the leakage of cytosolic proteins such as luciferase and lactate dehydrogenase and finally leads to the cell lysis (Mecke et al. 2005; Chen et al. 2004).

In a similar study, varying concentrations of ([G4]-PGLSA-OH)2-PEG3400 were incubated for 0.5 or 2 h with HT-29 human colon cancer cells. No cytotoxic effects were observed (Morgan et al. 2003). Jevprasesphant et al. studied the cytotoxicity of PAMAM dendrimers in Caco-2 cells; the results showed that anionic or half generation dendrimers had significantly lower toxicity than the cationic ones (Jevprasesphant et al. 2003).

Stability in the biological media is one of the crucial properties of IONPs in biomedical applications, since this is directly related to their diagnostic and therapeutic characteristic such as circulation time (Montazerabadi et al. 2015). In this study, to provide stabilized G_4_@IONPs, mPEG_4000_ coating was used, which possessed several advantages in aqueous solution (Longmire et al. 2008). The results indicated that the turbidity of G_4_@IONPs suspension after 72 h was equivalent to the turbidity of control suspension (FBS); the optical density (OD) enhancement of G_4_@IONPs suspension could be the result of instability of FBS solution, considering the increasing of FBS turbidity over the time.

The viability of MCF_7_ cells treated with G_4_@IONPs was reduced significantly after AMF exposure (*P* < 0.05); the concentration of G_4_@IONPs used in in vitro experiments was 500 µg/ml, which had shown no any cytotoxic effect. TUNEL outcomes also confirmed the MTT results and in a way that apoptotic cancer cells in HT + NPs group was significantly higher than that in control group (*P* > 0.05). The viability of HDF_1_ cells cultured with G_4_@IONPs did not decrease significantly due to AMF exposure (*P* > 0.05). This could be because of that normal cells are less sensitive to the magnetic hyperthermia than cancer cells (Oei et al. 2017).

It is worthful to compare our result with other studies outcomes in which other hyperthermia methods like photothermal and microwave-induced photodynamic therapy were used. Magnetic hyperthermia is not the only localized hyperthermia modality using NPs. Li et al. studied photothermal ablation of cervical cancer HeLa cells with copper sulfide (CuS) nanoparticles and near-infrared (NIR) laser beam at 808 nm. Their results showed that the photothermal destruction of HeLa cells occurred in a laser dose- and NP-concentration-dependent manner (Li et al. 2010). In another study, Yao et al. presented microwave-induced photodynamic therapy and applied copper cysteamine (Cu–Cy) NPs as a new type of photosensitizer. The outcomes revealed that microwave activation of Cu–Cy method could significantly demolish rat osteosarcoma cell line (UMR 106-01) in both in vitro and in vivo studies (Yao et al. 2016). In photodynamic therapy, other types of photosensitizer were investigated such as graphitic-phase carbon nitride (g-C_3_N_4_) quantum dots (QDs). The results of live/dead staining and flow cytometry in Chu et al. study showed that g-C3N4 QD-based photodynamic therapy could effectively kill cancer cells and promoted tumor cell death (Chu et al. 2017).

Our experimental data clearly indicated that G_4_@IONPs had the ability to increase *r*_2_ to 139.12 s^−1^mMol^−1^. Indeed, IONPs create a magnetic field around themselves and, thereby, generate small field inhomogeneities in the external magnetic field. Thus, *T*_2_ relaxivity time decreases due to the rapid dephasing of the spins (Li et al. 2013). The liver MR signal became progressively weaker post-intravenous injection of G_4_@IONPs when compared to that of the control mice before injection. The results indicated that G_4_@IONPs can be used as an MRI contrast agent.

## Conclusion

The current study indicated that G_4_@IONPs are promising therapeutic agents for magnetic hyperthermia of breast cancer cells; in addition, MR-imaging results showed that the synthesized nanocomposite is a capable MRI contrast agent for T_2_-weighted imaging, both in vitro and in vivo.

## Abbreviations

MRI: magnetic resonance imaging
G_4_@IONPs: fourth-generation dendrimer-coated iron-oxide nanoparticles
G_4_: fourth generation
PAMAM: polyamidoamine
SAR: specific absorption rate
NPs: nanoparticles
MNPs: magnetic nanoparticles
VSM: vibrating sample magnetometer
TEM: transmission electron microscopy
DLS: dynamic light scattering
XRD: X-ray diffraction
DMSO: dimethyl sulfoxide
RBCs: red blood cells
FBS: fetal bovine serum
ICP-MS: inductively coupled plasma mass spectrometry
SE: spin echoes
TE: echo time
TR: repetition times
FOV: field of view
SI: signal intensity
V_H_: hydrodynamic diameter
AMF: alternating magnetic field
*τ*_B_: Brownian relaxation time
OD: optical density
NIR: near-infrared
QDs: quantum dots

## References

[CR1] Barick KC, Singh S, Jadhav NV, Bahadur D, Pandey BN, Hassan PA (2012). pH-responsive peptide mimic shell cross-linked magnetic nanocarriers for combination therapy. Adv Func Mater.

[CR2] Barick K, Singh S, Bahadur D, Lawande MA, Patkar DP, Hassan P (2014). Carboxyl decorated Fe_3_O_4_ nanoparticles for MRI diagnosis and localized hyperthermia. J Colloid Interface Sci.

[CR3] Bordelon DE, Cornejo C, Grüttner C, Westphal F, DeWeese TL, Ivkov R (2011). Magnetic nanoparticle heating efficiency reveals magneto-structural differences when characterized with wide ranging and high amplitude alternating magnetic fields. J Appl Phys.

[CR4] Chandra S, Dietrich S, Lang H, Bahadur D (2011). Dendrimer–doxorubicin conjugate for enhanced therapeutic effects for cancer. J Mater Chem.

[CR5] Chen H-T, Neerman MF, Parrish AR, Simanek EE (2004). Cytotoxicity, hemolysis, and acute in vivo toxicity of dendrimers based on melamine, candidate vehicles for drug delivery. J Am Chem Soc.

[CR6] Chu X, Li K, Guo H, Zheng H, Shuda S, Wang X (2017). Exploration of graphitic-C_3_N_4_ quantum dots for microwave-induced photodynamic therapy. ACS Biomater Sci Eng..

[CR7] Deatsch AE, Evans BA (2014). Heating efficiency in magnetic nanoparticle hyperthermia. J Magn Magn Mater.

[CR8] Domingos RF, Baalousha MA, Ju-Nam Y, Reid MM, Tufenkji N, Lead JR (2009). Characterizing manufactured nanoparticles in the environment: multimethod determination of particle sizes. Environ Sci Technol.

[CR9] Etheridge M, Manuchehrabadi N, Franklin R, Bischof J. Superparamagnetic iron oxide nanoparticle heating: a basic tutorial. in: Nanoparticle Heat Transfer and Fluid Flow. CRC Press; 2012. p. 97–121.

[CR10] Feng W, Nie W, He C, Zhou X, Chen L, Qiu K (2014). Effect of pH-responsive alginate/chitosan multilayers coating on delivery efficiency, cellular uptake and biodistribution of mesoporous silica nanoparticles based nanocarriers. ACS Appl Mater Interfaces.

[CR11] Hong S, Leroueil PR, Janus EK, Peters JL, Kober M-M, Islam MT (2006). Interaction of polycationic polymers with supported lipid bilayers and cells: nanoscale hole formation and enhanced membrane permeability. Bioconjug Chem.

[CR12] Jevprasesphant R, Penny J, Jalal R, Attwood D, McKeown N (2003). D’emanuele A. The influence of surface modification on the cytotoxicity of PAMAM dendrimers. Int J Pharm.

[CR13] Jiang Q, Zheng S, Hong R, Deng S, Guo L, Hu R (2014). Folic acid-conjugated Fe_3_O_4_ magnetic nanoparticles for hyperthermia and MRI in vitro and in vivo. Appl Surf Sci.

[CR14] Julian JM, Brezinski DR. An infrared spectroscopy atlas for the coatings industry. 1991.

[CR15] Kalber TL, Smith CJ, Howe FA, Griffiths JR, Ryan AJ, Waterton JC (2005). A longitudinal study of R2* and R2 magnetic resonance imaging relaxation rate measurements in murine liver after a single administration of 3 different iron oxide-based contrast agents. Invest Radiol.

[CR16] Khodadust R, Unsoy G, Yalcın S, Gunduz G, Gunduz U (2013). PAMAM dendrimer-coated iron oxide nanoparticles: synthesis and characterization of different generations. J Nanopart Res.

[CR17] Khot V, Salunkhe A, Thorat N, Ningthoujam R, Pawar S (2013). Induction heating studies of dextran coated MgFe_2_O_4_ nanoparticles for magnetic hyperthermia. Dalton Trans.

[CR18] Kim H-A, Seo J-K, Kim T, Lee B-T (2014). Nanometrology and its perspectives in environmental research. Environ Health Toxicol.

[CR19] Kojima C, Turkbey B, Ogawa M, Bernardo M, Regino CA, Bryant LH (2011). Dendrimer-based MRI contrast agents: the effects of PEGylation on relaxivity and pharmacokinetics. Nanomed Nanotechnol Biol Med.

[CR20] Li Y, Lu W, Huang Q, Li C, Chen W (2010). In vitro photothermal ablation of tumor cells with CuS nanoparticles. Nanomedicine..

[CR21] Li L, Jiang W, Luo K, Song H, Lan F, Wu Y (2013). Superparamagnetic iron oxide nanoparticles as MRI contrast agents for non-invasive stem cell labeling and tracking. Theranostics..

[CR22] Li T, Shen X, Chen Y, Zhang C, Yan J, Yang H (2015). Polyetherimide-grafted Fe_3_O_4_@ SiO_2_ nanoparticles as theranostic agents for simultaneous VEGF siRNA delivery and magnetic resonance cell imaging. Int J Nanomed.

[CR23] Longmire M, Choyke PL, Kobayashi H (2008). Dendrimer-based contrast agents for molecular imaging. Curr Top Med Chem.

[CR24] Mamiya H, Jeyadevan B (2011). Hyperthermic effects of dissipative structures of magnetic nanoparticles in large alternating magnetic fields. Sci Rep..

[CR25] Mecke A, Majoros IJ, Patri AK, Baker JR, Banaszak Holl MM, Orr BG (2005). Lipid bilayer disruption by polycationic polymers: the roles of size and chemical functional group. Langmuir.

[CR26] Mecke A, Lee D-K, Ramamoorthy A, Orr BG, Banaszak Holl MM (2005). Synthetic and natural polycationic polymer nanoparticles interact selectively with fluid-phase domains of DMPC lipid bilayers. Langmuir.

[CR27] Mohammad F, Yusof NA (2014). Doxorubicin-loaded magnetic gold nanoshells for a combination therapy of hyperthermia and drug delivery. J Colloid Interface Sci.

[CR28] Montazerabadi AR, Oghabian MA, Irajirad R, Muhammadnejad S, Ahmadvand D, Delavari HH (2015). Development of gold-coated magnetic nanoparticles as a potential MRI contrast agent. NANO.

[CR29] Morgan MT, Carnahan MA, Immoos CE, Ribeiro AA, Finkelstein S, Lee SJ (2003). Dendritic molecular capsules for hydrophobic compounds. J Am Chem Soc.

[CR30] Natividad E, Castro M, Mediano A (2008). Accurate measurement of the specific absorption rate using a suitable adiabatic magnetothermal setup. Appl Phys Lett.

[CR31] Natividad E, Castro M, Mediano A (2009). Adiabatic vs. non-adiabatic determination of specific absorption rate of ferrofluids. J Magn Magn Mater.

[CR32] Oei A, Vriend L, Krawczyk P, Horsman M, Franken N, Crezee J (2017). Targeting therapy-resistant cancer stem cells by hyperthermia. Int J Hyperth.

[CR33] Pearce J, Giustini A, Stigliano R, Hoopes PJ (2013). Magnetic heating of nanoparticles: the importance of particle clustering to achieve therapeutic temperatures. J Nanotechnol Eng Med..

[CR34] Prasad N, Rathinasamy K, Panda D, Bahadur D (2007). Mechanism of cell death induced by magnetic hyperthermia with nanoparticles of γ-Mn x Fe_2_–x O_3_ synthesized by a single step process. J Mater Chem.

[CR35] Rosensweig RE (2002). Heating magnetic fluid with alternating magnetic field. J Magn Magn Mater.

[CR36] Salimi M, Shahbazi-Gahrouei D, Karbasi S, Kermani S, Razavi S (2013). Effect of extremely low-frequency (50 Hz) field on proliferation rate of human adipose-derived mesenchymal stem cells. J Isfahan Med School.

[CR37] Salimi M, Sarkar S, Fathi S, Alizadeh AM, Saber R, Moradi F (2018). Biodistribution, pharmacokinetics, and toxicity of dendrimer-coated iron oxide nanoparticles in BALB/c mice. Int J Nanomed.

[CR38] Samanta B, Yan H, Fischer NO, Shi J, Jerry DJ, Rotello VM (2008). Protein-passivated Fe_3_O_4_ nanoparticles: low toxicity and rapid heating for thermal therapy. J Mater Chem.

[CR39] Seib FP, Jones AT, Duncan R (2007). Comparison of the endocytic properties of linear and branched PEIs, and cationic PAMAM dendrimers in B16f10 melanoma cells. J Control Release.

[CR40] Tajabadi M, Khosroshahi ME, Bonakdar S (2013). An efficient method of SPION synthesis coated with third generation PAMAM dendrimer. Colloids Surf A.

[CR41] Tomalia DA (2006). Dendrimers as multi-purpose nanodevices for oncology drug delivery and diagnostic imaging. Nanomed Nanotechnol Biol Med.

[CR42] Tomalia DA, Reyna L, Svenson S (2007). Dendrimers as multi-purpose nanodevices for oncology drug delivery and diagnostic imaging.

[CR43] Tsubokawa N, Takayama T (2000). Surface modification of chitosan powder by grafting of ‘dendrimer-like’ hyperbranched polymer onto the surface. React Funct Polym.

[CR44] Wolinsky JB, Grinstaff MW (2008). Therapeutic and diagnostic applications of dendrimers for cancer treatment. Adv Drug Deliv Rev.

[CR45] Xia T, Kovochich M, Liong M, Meng H, Kabehie S, George S (2009). Polyethyleneimine coating enhances the cellular uptake of mesoporous silica nanoparticles and allows safe delivery of siRNA and DNA constructs. ACS Nano.

[CR46] Yamaura M, Camilo R, Sampaio L, Macedo M, Nakamura M, Toma H (2004). Preparation and characterization of (3-aminopropyl) triethoxysilane-coated magnetite nanoparticles. J Magn Magn Mater.

[CR47] Yao M, Ma L, Li L, Zhang J, Lim RX, Chen W (2016). A new modality for cancer treatment—nanoparticle mediated microwave induced photodynamic therapy. J Biomed Nanotechnol.

